# The human *EDAR* 370V/A polymorphism affects tooth root morphology potentially through the modification of a reaction–diffusion system

**DOI:** 10.1038/s41598-021-84653-4

**Published:** 2021-03-04

**Authors:** Keiichi Kataoka, Hironori Fujita, Mutsumi Isa, Shimpei Gotoh, Akira Arasaki, Hajime Ishida, Ryosuke Kimura

**Affiliations:** 1 Department of Human Biology and Anatomy, Graduate School of Medicine, University of the Ryukyus, Okinawa, 903-0215 Japan; 2 Department of Oral and Maxillofacial Functional Rehabilitation, Graduate School of Medicine, University of the Ryukyus, Okinawa, Japan; 3 Astrobiology Center, National Institutes of Natural Sciences, Tokyo, Japan; 4 National Institute for Basic Biology, National Institutes of Natural Sciences, Aichi, Japan; 5 Department of Basic Biology, School of Life Science, SOKENDAI (The Graduate School for Advanced Studies), Aichi, Japan

**Keywords:** Computational models, Genetic association study, Morphogenesis

## Abstract

Morphological variations in human teeth have long been recognized and, in particular, the spatial and temporal distribution of two patterns of dental features in Asia, i.e., Sinodonty and Sundadonty, have contributed to our understanding of the human migration history. However, the molecular mechanisms underlying such dental variations have not yet been completely elucidated. Recent studies have clarified that a nonsynonymous variant in the ectodysplasin A receptor gene (*EDAR* 370V/A; rs3827760) contributes to crown traits related to Sinodonty. In this study, we examined the association between the *EDAR* polymorphism and tooth root traits by using computed tomography images and identified that the effects of the *EDAR* variant on the number and shape of roots differed depending on the tooth type. In addition, to better understand tooth root morphogenesis, a computational analysis for patterns of tooth roots was performed, assuming a reaction–diffusion system. The computational study suggested that the complicated effects of the *EDAR* polymorphism could be explained when it is considered that EDAR modifies the syntheses of multiple related molecules working in the reaction–diffusion dynamics. In this study, we shed light on the molecular mechanisms of tooth root morphogenesis, which are less understood in comparison to those of tooth crown morphogenesis.

## Introduction

Morphological variations in human teeth have been well studied in the field of dental anthropology^1,2^. Previous studies have revealed intra- and inter-population diversity in tooth morphology. In Asian populations, some dental characteristics can be grouped as a composite phenotype referred to as “Mongoloid dental complex” by Hanihara^3^. Turner^4–6^ dichotomized the dental patterns seen in Asian populations and named these patterns Sinodonty and Sundadonty. Typical characteristics of Sinodonty are shoveling and double shoveling of upper central incisors (UI1s), single-rooted upper first premolars (UP1s), enamel extensions of upper first molars (UM1s), three-rooted lower first molars (LM1s), and lower second molars (LM2s) with five or more-cusps and a C-shaped single root. In contrast, Sundadonty tends to have two-rooted UP1s, two-rooted LM1s, and four-cusped and two-rooted LM2s.

Previous genetic studies have identified that a nonsynonymous polymorphism in the ectodysplasin A receptor (*EDAR*) gene (rs3827760; 370V/A or 1540T/C according to the amino acid or nucleotide sequence, respectively) is strongly associated with the Sinodonty and Sundadonty tooth crown characteristics, such as incisor shoveling and double shoveling, the number of LM2 cusps, as well as crown size^7–9^. This polymorphism has also been shown to have pleiotropic effects on the morphology of many organs, e.g., on hair thickness and straightness^10–13^, beard thickness^14^, eccrine sweat gland density^15^, earlobe shape^16^, and mandibular morphology^17,18^. In addition, a knock-in mouse model has shown that the human 370A allele is associated with a higher branch density of mammary glands and smaller mammary fat pads when compared to the 370V allele^15^. The EDAR 370A protein has been considered to be hyperfunctional: in vitro transfection assays have demonstrated that EDAR 370A activates nuclear factor-κB (NF-κB)-dependent gene expression at approximately double the level of EDAR 370V^19,20^. Regarding the molecular evolution, the 370A allele is the derived allele, but it is highly frequent in Asian and Native American populations and is absent or rare in African and European populations. Moreover, population genetics studies have suggested that a strong positive selection acted on the 370A allele in East Asia^10,15,21,22^.

EDAR and its ligand, ectodysplasin A (EDA), play an important role in the development of ectodermal derivatives, including skin appendages and teeth. In humans, dysfunctional mutations in *EDA*, *EDAR*, and *EDAR-associated death domain* (*EDARDD*) cause ectodermal dysplasia, a genetic disorder characterized by malformation of ectodermal structures, such as skin, hair, teeth, and sweat glands^23–26^. Similar phenotypes have been observed in the *Eda* and *Edar* mutant mice, tabby and downless^24,27–29^. The functions of the Eda/Edar pathway have been clarified through studies on the development of hair, feathers, and teeth using animal models. These ectodermal organs share common developmental mechanisms in which interactions between two adjacent tissue layers, the epithelium and mesenchyme, play key roles^30,31^. Eda and Edar are coexpressed throughout the ectoderm before the initiation of tooth development^32–34^. During the development of molar teeth in mice, Edar is expressed in the ectodermal signaling centers, i.e., in the placode and in the primary enamel knot at the cap stage^32,33,35^. It has been demonstrated that transgenic mice overexpressing Eda or Edar show molar teeth with extra cusps and, in some cases, supernumerary teeth^36,37^.

It has been indicated that the morphogenesis of teeth, as well as hair and feathers, can be explained by the epithelial–mesenchymal interactions and the reaction–diffusion dynamics in which reciprocal interactions between diffusible molecules form a specific morphological pattern in a self-organizing manner^38–40^. Regarding hair development, it has been shown that Wnt family members (WNTs) and Dickkopf WNT signaling pathway inhibitors (DKKs) determine hair follicle spacing through an activator–inhibitor system, one of the simplest reaction–diffusion models^41^. In tooth development, the activities of a few signaling molecules, such as WNTs, sonic hedgehog (SHH), fibroblast growth factors (FGFs), and bone morphogenetic proteins (BMPs), are reiterated over the course of the determinations of the dental arch region, tooth identity, and cusp number and positions^42,43^. These and other related molecules have thus been considered as candidates of the substances in the activator–inhibitor system working in tooth development. In molars, the shape is mainly determined by the pattern of secondary enamel knots that appear at the sites of the future cusps. Salazar-Ciudad and Jernvall^44^ have demonstrated that a reaction–diffusion model predicts the course of tooth morphogenesis and the cusp pattern variation among different mammalian species. It has been suggested that EDAR, positively modifying expression of activator and inhibitor molecules, is involved in the reaction–diffusion dynamics^40,45,46^.

The molecular mechanisms of the late stages of tooth development, including root formation, are less understood in comparison to those of the early stages leading to crown formation^47,48^. Previous studies have suggested that, in the process of tooth root bifurcation, mesenchymal cells actively proliferate in the root-forming areas and the Hertwig’s epithelial root sheath (HERS), interacting with mesenchymal cells, invaginates into the furcation areas^47,49^. However, it has not been fully elucidated what factors generate such cell patterns and determine the number and shape of tooth roots. Especially, the morphogenesis of C-shaped root remains unclear. *Eda* and *Edar* mutant mice (tabby and downless, respectively) display a high incidence of taurodontism or delay in bifurcation^50^, which indicates that the Eda/Edar pathway has an essential role in the root bifurcation. It has also been reported that EDAR is expressed in HERS^50^. In the present study, we examined the association of the *EDAR* 370V/A polymorphism with the number and shape of human tooth roots by analyzing computed tomography (CT) images. In addition, we performed a computational analysis assuming a reaction–diffusion model to infer how the *EDAR* polymorphism changes root morphology.

## Results

### The association of EDAR V370A with tooth root morphology

**Figure 1:**
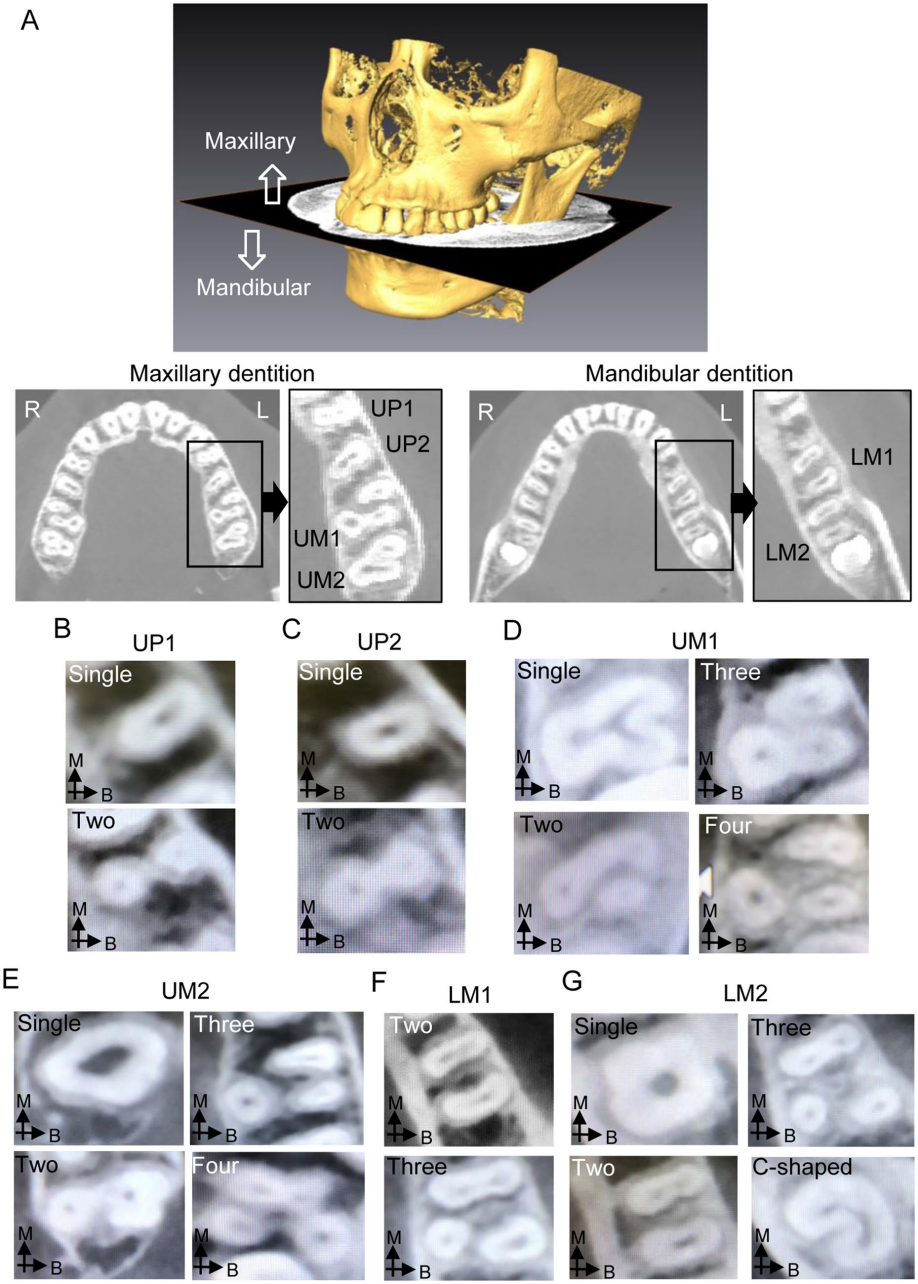
Observation of tooth root morphology using CT. (**A**) Reconstruction of the three-dimensional surface and the occlusal plane (OP) using Amira ver 6.0.0. The number and shape of roots in the maxillary and mandibular teeth were observed in slice images parallel to the OP. (**B**–**G**) Examples of the tooth root morphology observed in UP1s (**B**), UP2s (**C**), UM1s (**D**), UM2s (**E**), LM1s (**F**), and LM2s (**G**). The arrows indicate the buccal (**B**) and mesial (**M**) directions.

In the observation of maxillary tooth roots, we found single and two roots in UP1s and UP2s, and single, two, three, and four roots in UM1s and UM2s (Fig. 1 and Supplementary Table S2). In some individuals, the UP2s on both sides were congenitally absent (2.6%). The majority of individuals had single-rooted UP2s (91.2%), three-rooted UM1s (97.8%), and three-rooted UM2s (86.4%) on both sides. For UP1s, the major phenotype was single roots on both sides (65.1%), but individuals with two or more roots were also commonly observed. Regarding the mandibular tooth roots, we observed individuals with two-rooted (75.3%) and three-rooted (19.2%) LM1s on both sides, and some had a heterogeneous (two/three) phenotype (5.4%) (Fig. 1 and Supplementary Table S3). The most frequent phenotype for LM2s was two roots on both sides (56.5%), but individuals with C-shaped roots were also commonly observed.

In the genotyping of 255 individuals for *EDAR* V370A, we found 25V/V homozygotes (9.0%), 120V/A heterozygotes (47.1%), and 112 A/A homozygotes (43.9%). Using Fisher’s exact test, we observed significant associations between the *EDAR* genotype and the root shape of UP1s and LM2s (Table 1). Logistic regression analysis including age, sex, and region as covariates showed that the *EDAR* genotype was associated with the root shape of LM1s as well as UP1s and LM2s (Table 2). Interestingly, the 370A allele was associated with a decreased and increased number of UP1 and LM1 roots, respectively, while the allele was associated with the C-shaped root of LM2s. We also confirmed that there was no association between the region and tooth root morphology, but there was a significant association between age and the root number of UP1s.

**Table 1:** The *EDAR* 370V/A genotype and root morphology.

Tooth	Root shape	Total	Genotype			Fisher P
			V/V (%)	V/A (%)	A/A (%)	
**UP1**						
	1 (1|1)	155	12 (7.7)	63 (40.6)	80 (51.6)	0.010*
	2 ≤ (1|2, 2|2, 2|3)	83	9 (10.8)	48 (57.8)	26 (31.3)	
**UP2**						
	1 (1|1, 1|m)	208	18 (8.7)	97 (46.6)	93 (44.7)	0.91
	2 ≤ (1|2, 2|2, 1|3)	13	1 (7.7)	7 (53.8)	5 (38.5)	
**UM2**						
	≤ 2 (1|1, 1|2, 2|2, 1|3, 2|3)	29	1 (3.4)	16 (55.2)	12 (41.4)	0.47
	3 ≤ (3|3, 4|4)	207	21 (10.1)	94 (45.4)	92 (44.4)	
**LM1**						
	2 (2|2)	180	18 (10.0)	89 (49.4)	73 (40.6)	0.12
	3 (2|3, 3|3)	59	2 (3.4)	25 (42.4)	32 (54.2)	
**LM2**						
	C (2|C, C|C)	99	3 (3.0)	39 (39.4)	57 (57.6)	0.00087*
	2 (2|2)	134	15 (11.2)	72 (53.7)	47 (35.1)	

Root shape for each individual is indicated as a combination of both sides. 1: single root, 2: two roots, 3: three roots, 4: four roots, m: congenital missing, C: C-shaped root.

*Significant association (P < 0.05).

**Table 2:** Logistic regression analyses on the root shape of UP1s, LM1s, and LM2s.

Objective variable	Explanatory variables	Log odds ratio	Standard error	χ^2^	P
**UP1**					
Test: 1 (1|1) Ref: 2 ≤ (1|2, 2|2, 2|3)	Age	0.074	0.024	9.44	0.0021*
	Sex	0.520	0.302	2.96	0.087
	Region	0.096	0.089	1.16	0.28
	*EDAR* genotype	0.666	0.227	8.57	0.0034*
**LM1**					
Test: 3 (2|3, 3|3) Ref: 2 (2|2)	Age	− 0.043	0.026	2.72	0.099
	Sex	0.162	0.327	0.24	0.62
	Region	− 0.003	0.098	0.00	0.97
	*EDAR* genotype	0.529	0.256	4.27	0.039*
**LM2**					
Test: C (2|C, C|C) Ref: 2 (2|2)	Age	0.026	0.021	1.57	0.21
	Sex	0.558	0.296	3.55	0.060
	Region	0.087	0.091	0.91	0.34
	*EDAR* genotype	0.858	0.237	13.17	0.00028*

Phenotypes of each tooth are dichotomized into test and ref and the log odds ratio of test to ref is shown. *EDAR* genotype is represented as VV = 0, VA = 1 and AA = 2, sex as male = 0 and female = 1, and region as the number of grandparents originating from the Ryukyu Islands.

*Significant association (P < 0.05).

We also observed the tooth crown traits such as shoveling in UI1s, Carabelli cusp in UM1s, and the cusp numbers of UM2s and LM2s (Supplementary Table S4), and calculated the Spearman's rank correlation coefficients between dental traits (Table 3). Significant correlations were shown between the following pairs of traits: UP1 roots vs UP2 roots, UP1 roots vs UM2 roots, UP1 roots vs LM2 roots, UP2 roots vs LM2 roots, UP2 roots vs UI1 shoveling, UM1 roots vs UM2 roots, LM1 roots vs LM2 cusp number, LM2 roots vs UI1 shoveling, and UI1 shoveling vs LM2 cusp number. Compared with UI1 shoveling, UP1, LM1 and LM2 root traits were weakly, but significantly, correlated with the *EDAR* genotype (Table 3). Among the root traits that were significantly associated with the *EDAR* genotype, the LM2 root trait was significantly correlated with UI1 shoveling, but the UP1 and LM1 root traits were not. It is worth noting that the correlations between the crown and root traits of the same tooth (UM1, UM2, and LM2) were not significant. Additionally, regarding the root numbers of two adjacent teeth, a positive correlation was observed in UP1 vs UP2 and UM1 vs UM2, whereas a negative, but not significant, correlation was observed in UP2 vs UM1 and LM1 vs LM2.

**Table 3:** Spearman's rank correlation coefficients between dental traits.

Variables (ordered categories)	A	B	C	D	E	F	G	H	I	J	K
A. EDAR genotype [VV, VA, AA]	–	**4.1E − 03**	7.3E − 01	6.8E − 01	9.3E − 01	**3.7E − 02**	**3.4E − 04**	**6.0E − 13**	1.0E − 01	9.9E − 02	1.9E − 01
B. UP1 roots [1, 2≤]	**− 0.1855**	–	**1.5E − 04**	6.5E − 01	**6.9E − 05**	5.4E − 01	**2.3E − 04**	1.2E − 01	5.3E − 01	2.3E − 01	9.6E − 01
C. UP2 roots [1, 2≤]	− 0.0235	**0.2572**	–	1.3E − 01	1.7E − 01	3.9E − 01	**1.8E − 03**	**5.0E − 03**	8.5E − 01	5.8E − 01	4.8E − 01
D. UM1 roots [2, 3≤]	− 0.0278	0.0314	− 0.1072	–	**5.5E − 05**	3.0E − 01	7.4E − 01	5.8E − 01	5.1E − 01	6.2E − 01	7.6E − 01
E. UM2 roots [≤ 2, 3≤]	− 0.0061	**0.2632**	0.0951	**0.2721**	–	9.1E − 01	9.9E − 02	4.5E − 01	5.7E − 01	2.0E − 01	1.8E − 01
F. LM1 roots [2, 3]	**0.1351**	0.0412	− 0.0606	0.0717	0.0073	–	2.7E − 01	5.7E − 02	5.5E − 01	4.6E − 01	**2.7E − 02**
G. LM2 roots [C, 2]	**− 0.2327**	**0.2474**	**0.2186**	− 0.0236	0.1125	− 0.0725	–	**1.9E − 02**	4.7E − 01	8.1E − 02	2.1E − 01
H. UI1 Shoveling [≤ 2, 3≤]	**0.4571**	− 0.1074	**− 0.1999**	− 0.0393	− 0.0531	0.1296	**− 0.1616**	–	5.4E − 01	7.4E − 01	**2.2E − 02**
I. UM1 Carabelli cusp [0, 1]	− 0.1077	0.0427	− 0.0136	0.0461	0.0388	− 0.0406	0.0490	− 0.0410	–	2.5E − 01	4.8E − 01
J. UM2 cusp number [3, 4]	− 0.1085	0.0814	− 0.0389	− 0.0350	0.0873	− 0.0502	0.1188	− 0.0228	0.0767	–	8.8E − 02
K. LM2 cusp number [4, 5≤]	0.0898	0.0034	− 0.0515	0.0223	0.0948	**− 0.1537**	− 0.0891	**0.1613**	0.0497	0.1184	–

Lower left: Spearman's rank correlation coefficients. Upper right: P values.

Bold: significant correlation.

### Computer simulation assuming a reaction–diffusion model

**Figure 2:**
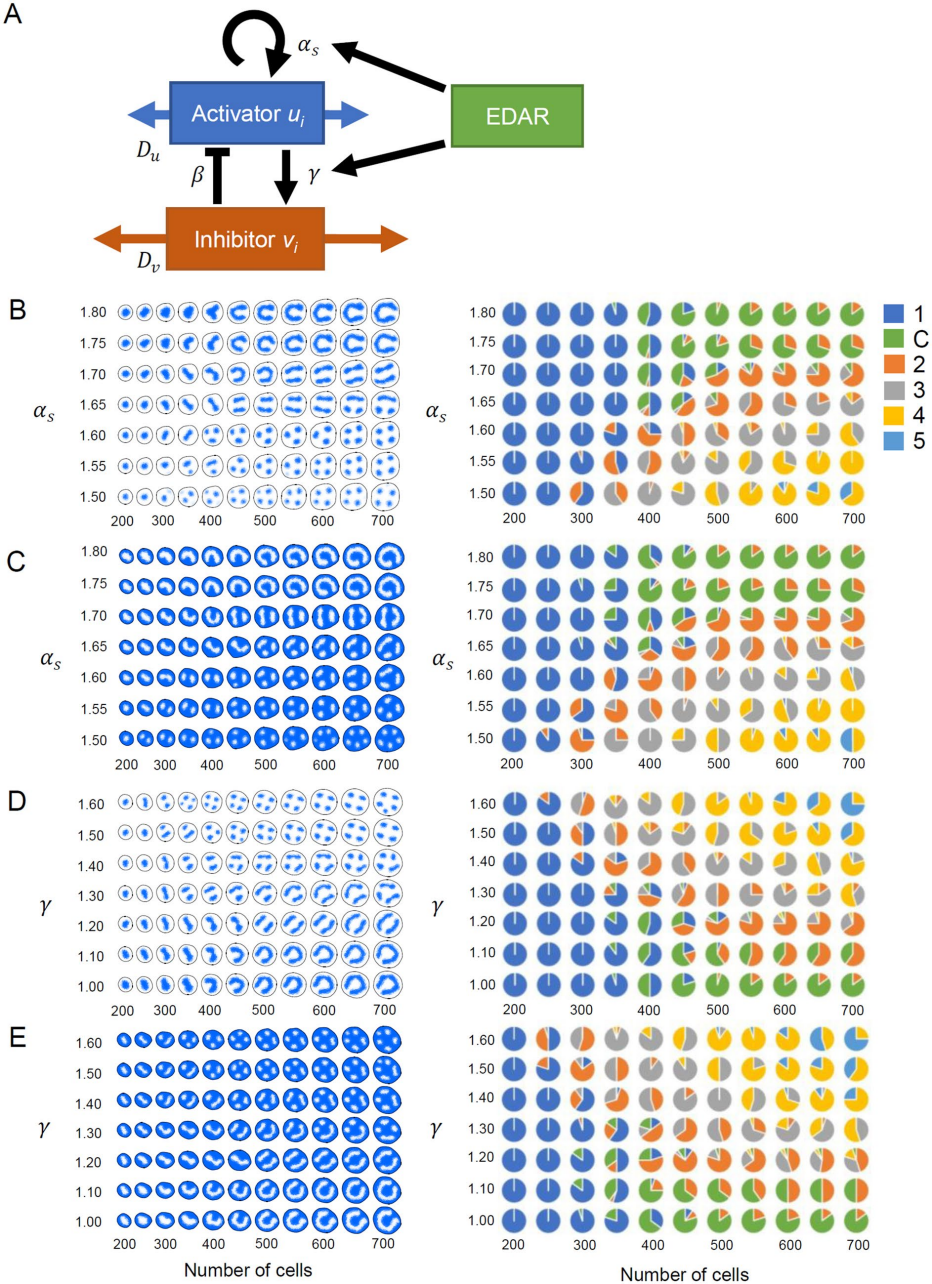
Computational analysis of tooth root morphogenesis assuming a reaction–diffusion system. (**A**) Schema of the activator–inhibitor system. Interactions between two diffusible substances, namely activator and inhibitor, generate a self-organized spatial pattern. In this simulation, the *EDAR* variant is assumed to be associated with the self activation of activator and the activation of inhibitor by activator. (**B**–**E**) A representative result (left) and the summary (right) of 20 independent simulations. The variation in the cell pattern is caused depending on *α*_*s*_ (strength of activator synthesis) (**B**, **C**) and *γ* (strength of inhibitor synthesis) (**D**, **E**). In addition, “spotted” and “reverse-spotted” patterns are emerged under the conditions that the maximum concentration of the activator is high (*u*_*max*_ = 10 *u*_*0*_; **B**, **D**) and low (*u*_*max*_ = 1.1 *u*_*0*_; **C**, **E**), respectively. The blue color indicates a high concentration of the activator. Detailed conditions for computer simulations are described in “Materials and methods”, and parameter values used are as follows: *α*_*s*_ = 1.5–1.8 and *γ* = 1.0 (**B**, **C**); *α*_*s*_ = 1.8 and *γ* = 1.0–1.6 (**D**, **E**); *α*_*m*_ = 1.0 (**B**, **D**) or − 0.1 (**C**, **E**); and *α*_*d*_ = 1.0, *β* = 1.0, *δ* = 1.0, *ε* = 1.0, *D*_*u*_ = 0.25, *D*_*v*_ = 0.5, and *u*_*0*_ = *δε*/[*βγ* − (*α*_*s*_ − *α*_*d*_) *δ*] (**B**–**E**).

**Figure 3:**
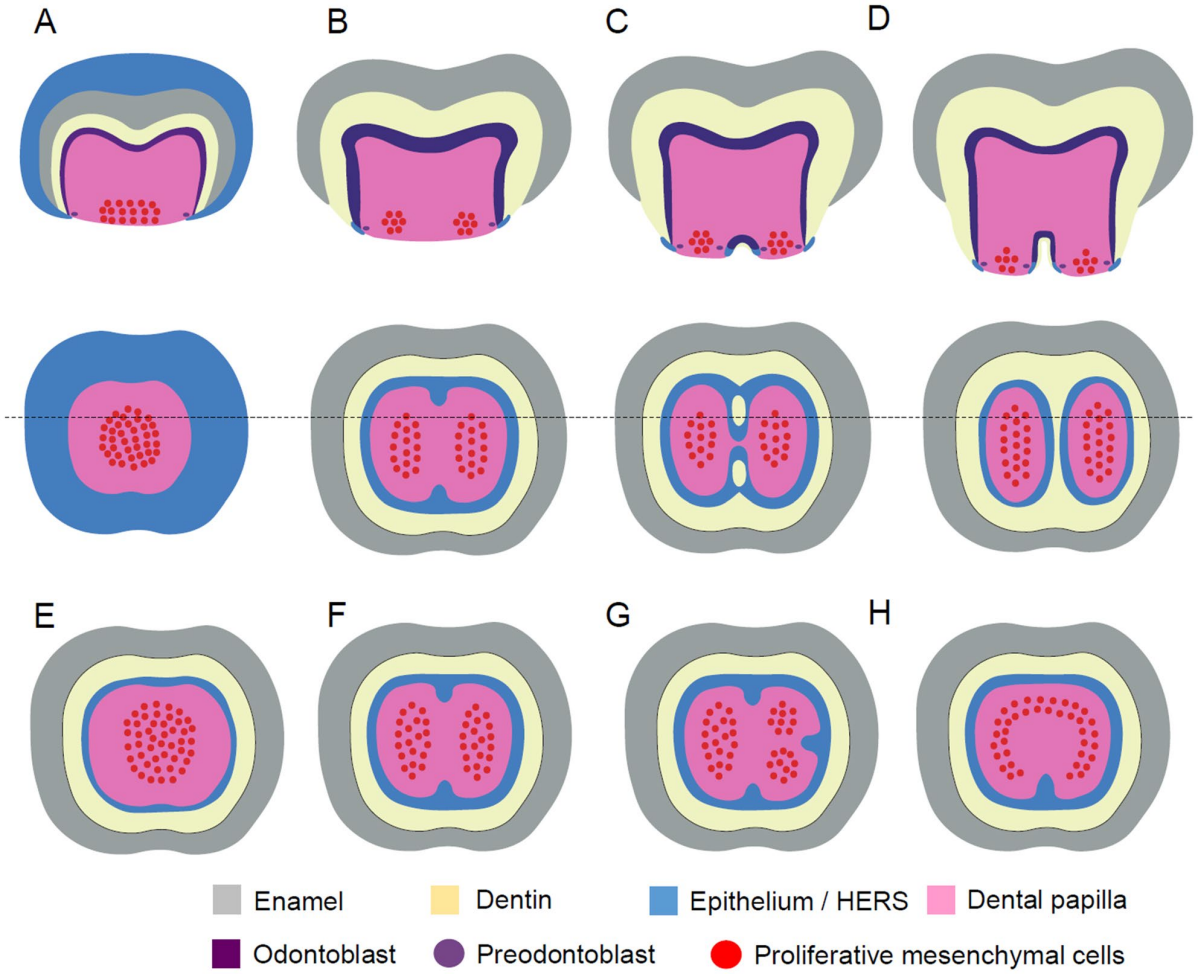
Schema of tooth root bifurcation. (**A**–**D**) Sagittal section and axial view of a developing human tooth at the stage of crown development (**A**) and at the stages of root development; before root bifurcation (**B**), during the dentin island formation (**C**), and after root bifurcation (**D**). The broken line denotes the sagittal section of the tooth. (**E**–**H**) Axial view of a developing tooth with one root (**E**), two roots (**F**), three roots (**G**), or a C-shaped root (**H**). The illustrations were created using Adobe Illustrator ver 24.

The effects of *EDAR* V370A on tooth root morphology cannot be interpreted in a simple way since it varies depending on tooth type. For further understanding of the effects of *EDAR* V370A, we performed a computer simulation for two-dimensional cell patterns assuming a reaction–diffusion model. In this simulation, we hypothesized that (1) the *EDAR* polymorphism positively modifies the induction of both activator and inhibitor in a pure reaction–diffusion system that determines the cell patterns in root development (Fig. 2A) and that (2) the areas that become future roots or future furcation areas in the apical surface of dental papilla are determined by the cell pattern at the beginning of root formation (Fig. 3). Our simplistic model can produce various spotted or reverse spotted patterns that can be interpreted as various root types, including one root, two roots, three roots, four roots, and C-shaped root (Fig. 2B–E and Supplementary Figs. S1–S4). The root shape pattern depends on parameters *α*_*s*_ and *γ*, which represent the strengths of induction for activator and inhibitor, respectively (Fig. 2B–E). Interestingly, *α*_*s*_ and *γ* have opposite effects; the root number increases as *α*_*s*_ decreases or *γ* increases, and C-shaped root patterns appear when *α*_*s*_ is high or *γ* is low. As the size of the area (i.e. total cell number) of the apical surface increases over time, the root number increases. In addition, the cell pattern can be flipped depending on *u*_*max*_, the saturation value of the activator. When the equilibrium value of the activator *u*_*0*_ is closer to 0 than to *u*_*max*_ (i.e. *u*_*max*_ = 10*u*_*0*_; Fig. 2B,D), areas where the activator is concentrated form spots. However, the pattern is reversed on the condition that *u*_*0*_ is closer to *u*_*max*_ than to 0 (i.e. *u*_*max*_ = 1.1*u*_*0*_; Fig. 2C,E). In case of spotted patterns, it can be interpreted that the activator in the reaction–diffusion system also functions as a signaling molecule that induces mesenchymal cell proliferations to form roots. In contrast, reverse-spotted patterns indicate that the activator functions as a molecule that causes the inhibition of mesenchymal cell proliferation and the promotion of furcation.

## Discussion

Previous studies have shown an association between the *EDAR* 370A allele and crown characteristics, such as incisor shoveling and double shoveling, an increased number of LM2 cusps, and an increased crown size, which are typical phenotypes in Sinodonty^7–9^. However, the association between the *EDAR* 370A allele and root characteristics has not yet been examined. In this study, we observed the root morphology using CT images and found that the *EDAR* 370A allele was significantly associated with the root phenotypes related to Sinodonty, i.e., single-rooted UP1s, three-rooted LM1s, and LM2s with a C-shaped single root. Worthy of attention here is that the effects of the *EDAR* 370A allele differed between the three tooth types: the 370A allele was associated with a decreased and increased number of roots in UP1s and LM1s, respectively, and an unseparated root in LM2s.

We confirmed that UI1 shoveling is strongly correlated with the *EDAR* genotype^7,8^. Although the LM2 cusp number had been reported to be associated with the *EDAR* genotype^8^, their correlation was not significant in the present study. This study showed that the associations of the *EDAR* genotype with UM2, LM1, and LM2 root traits are weaker than that with UI1 shoveling. In general, correlations among the *EDAR*-associated crown and root traits were lower than their correlations with the *EDAR* genotype (Table 3). In addition, no significant correlation was observed even between the crown and root traits of the same tooth. These results suggest how complicated the dental morphogenesis is.

In root formation, HERS is thought to play important roles^51–53^. HERS is derived from the epithelium at the cuff of enamel formation, which is referred to as the cervical loop. The first step of root formation occurs through the elongation of HERS and interactions between HERS and mesenchymal cells. Preodontoblasts are co-localized and make contact with HERS, and then differentiate into odontoblasts that secrete radicular dentin. In the multi-rooted teeth of mice, HERS shows tongue-shaped inward protrusions that join to form furcation^54–58^. However, the mechanisms of the HERS invagination into furcation areas have not fully elucidated. A recent study has suggested that tongue-shaped epithelial protrusions in the cervical loop, termed cervical tongues, appear before HERS is formed, and that the positions of the cervical tongues are partly dependent on the shape of the crown and the positions of cusps^59^. In addition, some have argued that the shape of the cervical loop and the direction of HERS elongation are passively determined by mesenchymal cell proliferation in the dental papilla. Mesenchymal cell proliferation is active in root-forming areas, but not in furcation areas where HERS can be elongated horizontally at the border between the dental papilla and the dental follicle^59–61^. In human and rat molars, unlike murine molars, the formation of dentin islands appears at the center of the furcation area in the dental papilla independently of the surrounding dentin^62–64^. Then, the furcation area is formed by fusion of dentin islands and the surrounding dentin.

In this study, we assumed that the root morphology is determined by self-organizing cell distributions in the apical surface of the dental papilla at the beginning of root formation (Fig. 3). Several patterns of root morphology, including the C-shaped root, could be reproduced by a pure reaction–diffusion model, in which we implemented the growth of the area of the apical surface but not the invagination of epithelial cells (Fig. 2). We recognize that our model is simplistic and the real system must be more complicated. To pursue realistic models, we need to consider growths, migrations and interactions of epithelial and mesenchymal cells and various interactions between multiple numbers of molecules. However, even a simple model can be expected to be useful for understanding the essential molecular dynamics in the tooth root morphogenesis. The inferences drawn from the simulation study were as follows: (1) spotted and reverse-spotted patterns can be produced depending on the saturation value of the activator (*u*_*max*_); (2) an increased size of the apical surface at the beginning of root formation is associated with an increased number of roots; (3) the strength of activator synthesis (*α*_*s*_) is negatively associated with the root number; and (4) the strength of inhibitor synthesis (*γ*) is positively associated with the root number.

So far, several signaling pathways, including Fgfs, Bmps, Shh, and Wnts, have been proposed to be involved in the reaction–diffusion system in studies of hair, feathers, and tooth crown^40,44,65^. In particular, the Wnt/β-catenin signaling pathway works in the reaction–diffusion system in hair follicle spacing^41^, and is thus a strong candidate in tooth root formation as well. In the initiation of root formation, Wnt10a and β-catenin are expressed in preodontoblasts, odontoblasts and HERS, which are distributed similar to the reverse-spotted pattern in our simulation^66–69^. Dkk1, which likely acts as the inhibitor in the reaction–diffusion system, is also expressed in these cells^68,70,71^. Therefore, the localizations of both Wnt10a and Dkk1 are consistent with our pure reaction–diffusion model under the condition of a low saturation value of the activator (*u*_*max*_) (Fig. 2C,E). Other supportive evidences are that Wnt10a is involved in dentinogenesis and mineralization through dentin sialophosphoprotein^66^, and that disruption of the Wnt/β-catenin pathway or overexpression of Dkk1 arrests root odontogenesis during tooth root development^69,71^. In addition, the Wnt/β-catenin pathway has been shown to inhibit dental pulp stem cell differentiation^72^. Another candidate is the system involving BMPs and FGFs^73^. Bmp4 is expressed in preodontoblasts around HERS^74^ and it regulates HERS elongation^75^. The real system is likely to be more complicated and to involve multiple signaling pathways and their dynamic interactions^40,48,58,76–78^. Further experimental evidence is required to support that tooth root morphogenesis is controlled through reaction–diffusion dynamics.

At the root formation stage, *Edar* expression appears in HERS^50^. Although we did not explicitly implement the HERS elongation in our simulation study, it is hypothesized that HERS invagenates depending on the pattern of signaling molecules and EDAR modifies the cell distribution through direct and indirect effects on these signaling molecules^79–86^. Our simulation results provide a possible explanation for the discrepancy in the effect of the *EDAR* 370A allele between tooth types. Given that the *EDAR* 370A allele positively modifies both activator and inhibitor syntheses, the net effect can depend on tooth type and determine whether the number of roots increases or decreases: it is possible that the *EDAR* 370A allele has a stronger influence on activator synthesis than on inhibitor synthesis in UP1s and LM2s, whereas the opposite happens in LM1s.

Additionally, the opposite *EDAR* 370A effects between LM1s and LM2s may be attributed to the partitioning of the areas for the two adjacent teeth. The first stage of tooth development is the formation of the dental lamina. Then, the dental region is partitioned into compartments for incisor, canine, premolar, and molar regions. Finally, these compartments are further partitioned to form each tooth^43^. In the development of molars, the dental placodes appear sequentially from anterior to posterior and, therefore, an increased size of LM1 may cause a decreased size of LM2 within a restricted space of the compartment. EDAR also participates in the development of the signaling centers of the placodes in molars^45^. In this study, we observed a negative correlation between LM1 and LM2 root numbers. However, the correlation is not significant and weaker than their correlations with the EDAR genotype. These results suggest that the area partitioning between LM1s and LM2s is not a major factor that affects their root morphology.

In conclusion, this study showed that the *EDAR* 370V/A polymorphism is associated with the root morphology of UP1s, LM1s, and LM2s, but its effects on the root number are not simple. The *EDAR* genotype is a strong genetic factor that is a determinant of the tooth morphology dimorphism observed in Asian populations, but there must be other genetic factors involved as well. Previous studies have demonstrated that common genetic variants of *WNT10A* and *PAX9* are associated with tooth crown size^87,88^, and these variants may thus also be associated with root morphology. The present study also demonstrated that the examination of the modulation of morphology by a common variant is useful for understanding the mechanism of morphogenesis. The molecular interactions that occur during tooth morphogenesis are not yet fully understood. However, the accumulation of data from continued efforts will enable the complete picture of the molecular mechanisms of tooth morphogenesis to be elucidated.

## Materials and methods

### Subjects

The subjects were 255 Japanese patients (98 males and 157 females; 20–69 years of age) who underwent cone-beam CT scanning (3D Accuitomo F17D, J. Morita Mfg. Corp., Kyoto, Japan) for the purpose of dental/oral surgery and treatments at the University of the Ryukyus Hospital. In a questionnaire, we asked for the birthplaces of their grandparents, i.e., either mainland Japan or the Ryukyu Islands (Supplementary Table S1). We excluded individuals who had any grandparent(s) originating from countries other than Japan. Plaster casts of permanent dentition were obtained from each subject. Saliva (2.0 ml) was collected and stored using Oragene DNA (DNA Genotek, Ottawa, Canada). We obtained written informed consent from the patients for their participation in this study. This study was approved by the research ethics committee of the University of the Ryukyus and all research was performed in accordance with relevant guidelines/regulations.

### Morphological analysis

Morphological analysis of the CT images (voxel size: 250 μm) was performed using the three-dimensional imaging software Amira ver 6.0.0 (Thermo Fisher Scientific, Waltham, USA). After volume rendering of the Digital Imaging and Communications in Medicine (DICOM) data, we defined the occlusal plane using the mesial contact between both sides of the maxillary central incisors and the mesial buccal cusps of the left and right first molars (Fig. 1). Since UP1s, UP2s, UM1s, UM2s, LM1s, and LM2s have variations in the root number and shape, we observed these teeth in slice images parallel to the occlusal plane at intervals of 1.0 mm. Based on the images, we counted the number of roots and categorized the tooth root phenotypes as single (1), two (2), three (3), four (4), and C-shaped (C) root(s), or congenital missing (m); the phenotype for each individual is indicated by a combination of the categories of both sides (Supplementary Tables S2 and S3). For each tooth type, when either the left or right tooth was absent due to tooth extraction or treatment, we excluded the case from the analysis of the root morphology.

Using the plaster casts from the subjects. we also observed four tooth crown traits (shoveling of UI1s, Carabelli cusp of UM1s, the cusp number of UM1, and the cusp number of LM2) according to the the Arizona State University dental anthropology system^89^. We observed both left and right teeth. When tooth was absent or unobservable on one side, the phenotype on the other side was used. When the phenotypes are different between the left and right teeth, the less frequent phenotype was adopted.

### SNP genotyping

DNA was extracted from saliva that had been collected and stored in Oragene DNA collection kits using standard methods. Genotyping for *EDAR* 370 V/A (T1540C; rs3827760) was performed using a Taqman Genotyping Assay (Thermo Fisher Scientific, Waltham, USA).

### Statistical analysis

To examine the association between the *EDAR* 370V/A genotype and tooth morphology, Fisher’s exact test and logistic regression analysis were performed using JMP (SAS Institute Japan Ltd., Tokyo, Japan). In these analyses, the phenotypes for each tooth were dichotomized into two classes by merging or excluding some phenotypes. For instance, when the tooth root shape differed between the left and right sides, the case was classified into the less common phenotype. As covariates in the logistic regression analysis, we included sex (male = 1; female = 2) and region. Region was defined as the number of grandparents originating from the Ryukyu Islands (0, 1, 2, 3, or 4; Supplementary Table S1). The *EDAR* 370V/A genotype is denoted by the number of the A allele (V/V = 0; V/A = 1; A/A = 2). Spearman's rank correlation and P values were calculated among the *EDAR* genotype, tooth root traits, and tooth crown traits.

### Computational analysis

The numerical calculations were implemented in C, and the graphics of cell networks were made in Mathematica (Wolfram Research Inc., Champaign, USA). In the numerical calculations, two-dimensional cell patterns were simulated based on repeated cycles of three steps: cell division, cell network dynamics, and reaction–diffusion dynamics^90^.

Throughout the calculation, the number of cells increased from 10 to 1000 cells. The cell network system used in this study was similar to that of Prusinkiewicz and Lindenmayer^91^, and has been described elsewhere^90^. Cells proliferate and move by outward turgor pressures as follows: polygonal cells are tightly arranged in a two-dimensional space and are separated from each other by a straight cell wall. At a regular time interval *T* = 50.0, the cell with the largest area divides by the cell wall that passes through the gravity center of the cell in a random direction. The position of vertex *i*, ${\mathbf{u}}_{i}=({x}_{i},{y}_{i})$, moves depending on the force acting on vertex *i* (${\mathbf{F}}_{i}$), which consists of elastic forces of cell walls connecting to the vertex (${\mathbf{F}}_{Sj}$) and turgor pressures of surrounding cells (${\mathbf{F}}_{Pj}$) (Supplementary Fig. S5). The vertex position changes according to the following equations:

$$\frac{d{\mathbf{u}}_{i}}{dt}={k}_{T}{\mathbf{F}}_{i}$$

$${\mathbf{F}}_{i}=\sum_{j}({\mathbf{F}}_{Sj}+{\mathbf{F}}_{Pj})$$

$${\mathbf{F}}_{Sj}={k}_{s}\left({l}_{j}-{l}_{0}\right)$$

$${\mathbf{F}}_{Pj}={\mathbf{P}}_{j1}+{\mathbf{P}}_{j2}$$

$$\left|{\mathbf{P}}_{jm}\right|={k}_{P}/{V}_{jm} (m=1, 2)$$

where ${\mathbf{F}}_{i}$ is the total force acting on vertex *i*; *j* indicates the cell wall connecting to vertex *i*; ${\mathbf{F}}_{Sj}$ is the elastic force of cell wall *j* acting on vertex *i*; ${l}_{j}$ is the length of cell wall *j*; ${l}_{0}$ is the constant that corresponds to the rest length of cell wall; ${\mathbf{F}}_{Pj}$ is the sum of ${\mathbf{P}}_{j1}$ and ${\mathbf{P}}_{j2}$, which are effects of turgor pressures acting on cell wall *j* from neighboring cell *j*1 and *j*2, respectively; the strengths of ${\mathbf{P}}_{j1}$ and ${\mathbf{P}}_{j2}$ are inverse proportional to *V*_*j*1_ and *V*_*j*2_ (i.e. areas of cell *j*1 and *j*2), respectively; and *k*_*T*_ = 0.5, *k*_*S*_ = 1.0, *l*_0_ = 0.3, and *k*_*P*_ = 1.0 are constants. Vertex position (i.e. cellular position) was calculated to the equilibrium state.

The reaction–diffusion dynamics of the activator (*u*_*i*_) and inhibitor (*v*_*i*_) in the *i*th cell is described by the following form of equations:

1a $$\frac{d{u}_{i}}{dt}={\varphi }\left(\varepsilon +{\alpha }_{s}{u}_{i}-\beta {v}_{i}\right)-\left[{\alpha }_{d}+\psi \right]{u}_{i}+{D}_{u}\sum_{j=neighbors}\left({u}_{j}-{u}_{i}\right)$$

1b $$\frac{d{v}_{i}}{dt}=\gamma {u}_{i}-\delta {v}_{i}+{D}_{v}\sum_{j=neighbors}\left({v}_{j}-{v}_{i}\right)$$

1c $${{\varphi }}\left( x \right) = \left\{ {\begin{array}{*{20}{l}} {0\left( {x < 0} \right)} \\ {x\left( {0 \leq x \leq {\alpha_d}{u_{max}}} \right)} \\ {{\alpha_d}{u_{max}}({\alpha_d}{u_{max}} < x)} \end{array}} \right.$$

1d $$\psi = \left\{ {\begin{array}{*{20}l} {\alpha _{m} \left( {{\text{in}}\;{\text{marginal}}\;{\text{cells}}} \right)} \hfill \\ {0\left( {{\text{in}}\;{\text{non - marginal}}\;{\text{cells}}} \right)} \hfill \\ \end{array} } \right.$$

where *α*_*s*_, *α*_*d*_, *α*_*m*,_ *β*, *γ*, *δ*, *ε*, *D*_*u*_, *D*_*v*_, and *u*_*max*_ are constants. Equations (1a) or (1b) includes terms for the synthesis, degradation, and diffusion of the activator or inhibitor, respectively. The activator is induced by itself in the strength *α*_*s*_ and repressed by the inhibitor in the intensity *β*. In contrast, the inhibitor is induced by the activator in the strength *γ*. The activator or inhibitor decays at the rate *α*_*d*_ or *δ*, respectively. *ψ* modifies the rate of degradation of the activator in marginal cells, which prevents spot patterns from appearing in the edge of cell networks. The activator or inhibitor diffuses between adjacent cells with the diffusion coefficient *D*_*u*_ or *D*_*v*_, respectively. The equilibrium values of *u*_*i*_ and *v*_*i*_ in non-marginal cells are described by ${u}_{0}=\delta \varepsilon /(\beta \gamma -({\alpha }_{s}-{\alpha }_{d})\delta )$ and ${v}_{0}=\gamma \varepsilon /(\beta \gamma -({\alpha }_{s}-{\alpha }_{d})\delta )$, respectively, and *u*_*max*_ (> *u*_*0*_) is the constant that corresponds to the saturation value of *u*_*i*_ (i.e. $0\le {u}_{i}\le {u}_{max}$). In each of the reaction–diffusion dynamics steps, numerical calculations using the Euler method were carried out until reaching an almost steady state when the total time *Td* = 50.0 and the time step *dt* = 0.02. The parameter values in the numerical calculations are described in Fig. 2. For each parameter set, we performed 20 independent calculations in which the initial values of variables were given as their equilibrium with a random fluctuation of 1.0%.

## Supplementary Information

Supplementary Information.
