# Supplementary material for: The human EDAR 370V/A polymorphism affects tooth root morphology potentially through the modification of a reaction–diffusion system
Source: Sci Rep. 2021 Mar 4;11:5143. doi: 10.1038/s41598-021-84653-4 (PMC7933414; doi:10.1038/s41598-021-84653-4)
Supplement: Supplementary file 1 — Supplementary Information. [file 41598_2021_84653_MOESM1_ESM.pdf]

The human *EDAR* 370V/A polymorphism affects tooth root morphology potentially through the modification of a reaction-diffusion system

Keiichi Kataoka, Hironori Fujita, Mutsumi Isa, Shimpei Gotoh, Akira Arasaki, Hajime Ishida, and Ryosuke Kimura

Supplementary Information

Supplementary Table S1.
Supplementary Table S2.
Supplementary Table S3.
Supplementary Table S4.
Supplementary Fig. S1.
Supplementary Fig. S2.
Supplementary Fig. S3.
Supplementary Fig. S4.
Supplementary Fig. S5.

Supplementary Table S1. Self-reported origins of the four grandparents of each subject.

Region (Mainland Japan/Ryukyu Islands)	The number of individuals
4/0	51
3/1	0
2/2	14
1/3	10
0/4	180

Supplementary Table S2. Tooth root phenotyping for upper premolars and molars.

Tooth	Root shape	Number of individuals
UP1		
n=238	1|1	155
	1|2	10
	2|2	72
	2|3	1
UP2		
n=227	1|1	207
	1|2	7
	2|2	5
	1|3	1
	1|m	1
	m|m	6
UM1		
n=224	1|1	1
	2|2	3
	3|3	219
	1|4	1
UM2		
n=236	1|1	8
	1|2	4
	2|2	11
	1|3	1
	2|3	5
	3|3	204
	3|4	3

UP1: Upper (maxillary) first premolars, UP2: Upper (maxillary) second premolars, UM1: Upper (maxillary) first molars, UM2: Upper (maxillary) second molars. Root shape is indicated as a combination of both sides. 1: single root, 2: two roots, 3: three roots, 4: four roots, m: congenital missing.

Supplementary Table S3. Tooth root phenotyping for lower molars.

Tooth	Root shape	Number of individuals
LM1		
n=239	2|2	180
	2|3	13
	3|3	46
LM2		
n=239	1|1	4
	2|2	135
	2|3	1
	2|C	22
	3|C	1
	C|C	76

LM1: Lower (mandibular) first molars, LM2: Lower (mandibular) second molars. Root shape is indicated as a combination of both sides. 1: single root, 2: two roots, 3: three roots, C: C-shaped root.

Supplementary Table S4. Tooth crown phenotyping.

Trait	Phenotype	Number of individuals
UI1 shoveling grade		
n=226	1	22
	2	52
	3	137
	4	14
	5	1
UM1 Carabelli cusp		
n=231	absent (0)	209
	present (1)	22
UM2 cusp number		
n=232	3	13
	4	219
LM2 cusp number		
n=215	4	41
	5≤	174

UI1: Upper (maxillary) central incisors, UM1: Upper (maxillary) first molars, UM2: Upper (maxillary) second molars, LM2: lower (mandibular) second molars. UI1 Shoveling grade (0-7) was judged according to the Arizona State University dental anthropology system (Turner et al. 1991).

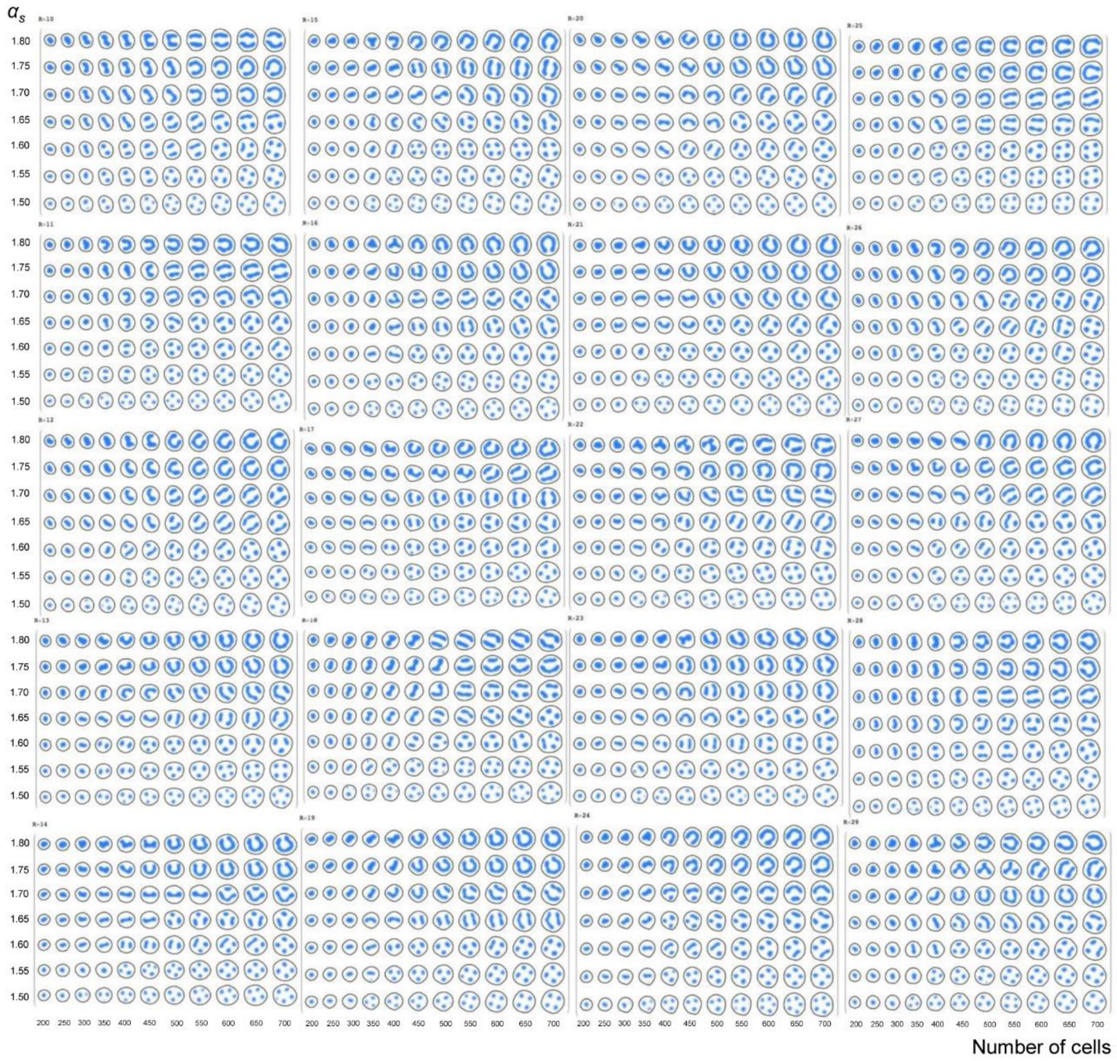

Supplementary Fig. S1. Computational analysis for various α_s values in the condition of u_{max} = 10u_0. Parameter values are the same as those in Fig. 2B. The results of 20 independent simulations are shown.

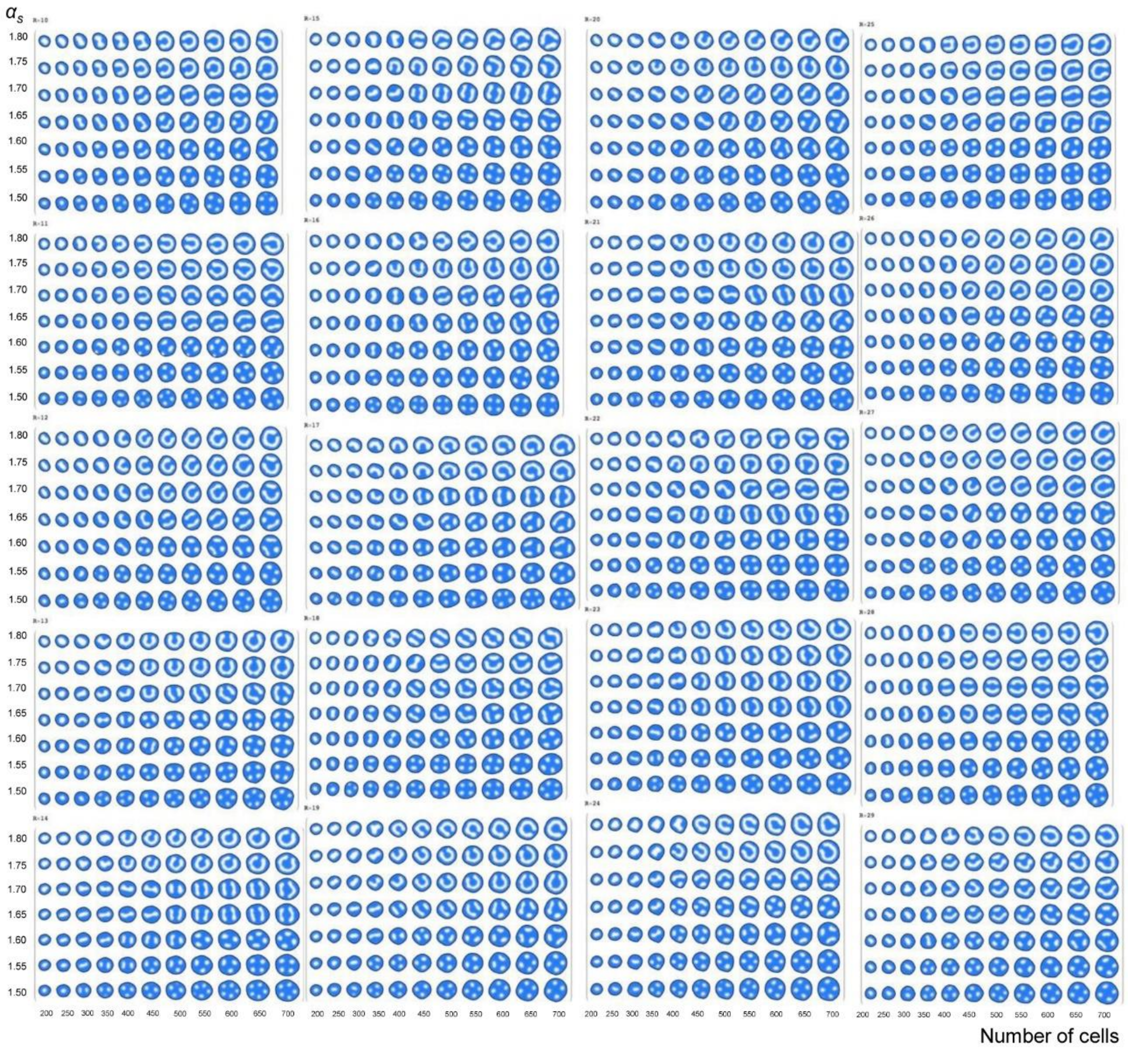

Supplementary Fig. S2. Computational analysis for various α_s values in the condition of u_{max} = 1.1u_0. Parameter values are the same as those in Fig. 2C. The results of 20 independent simulations are shown.

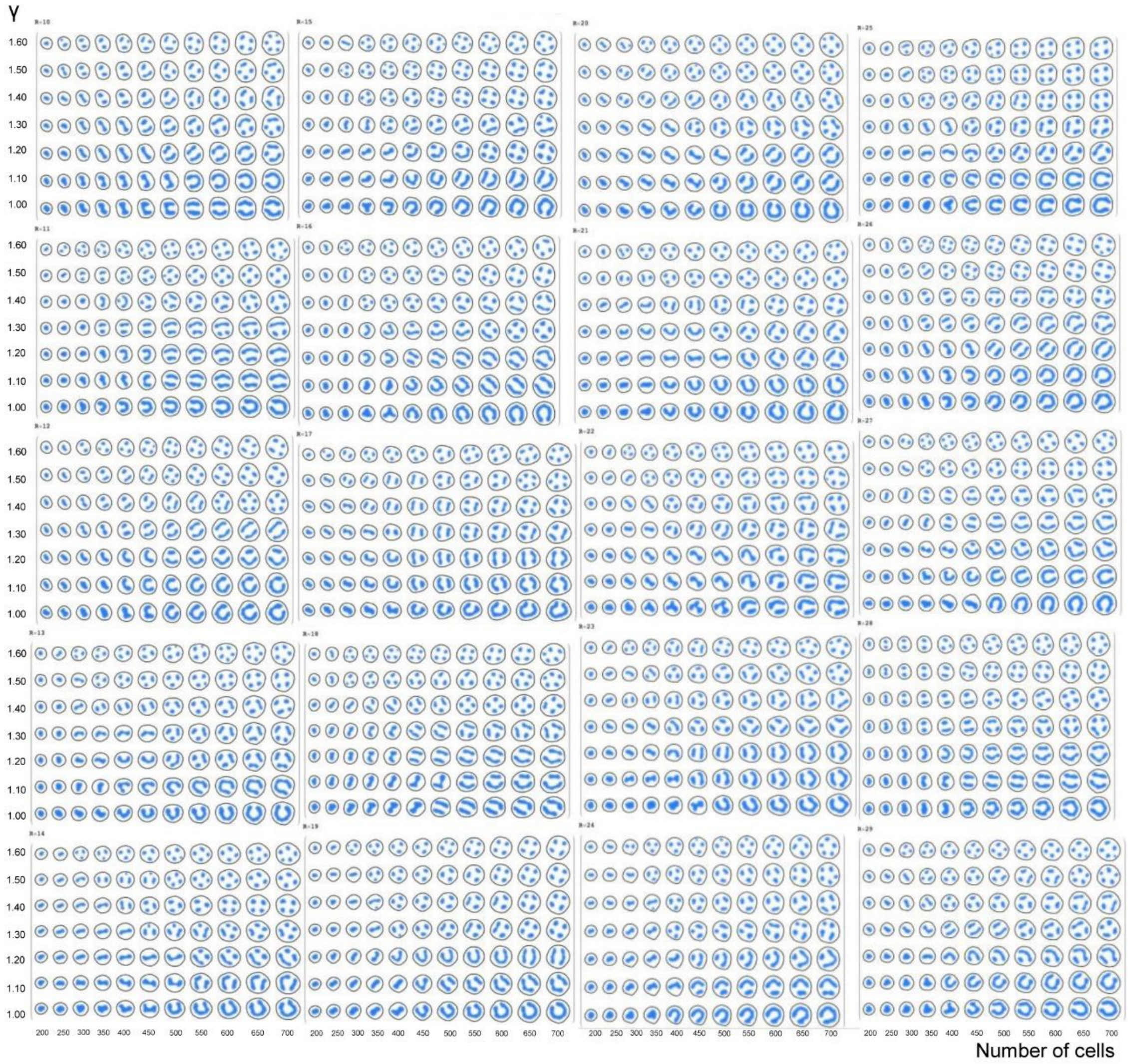

Supplementary Fig. S3. Computational analysis for various γ values in the condition of u_{max} = 10u_0. Parameter values are the same as those in Fig. 2D. The results of 20 independent simulations are shown.

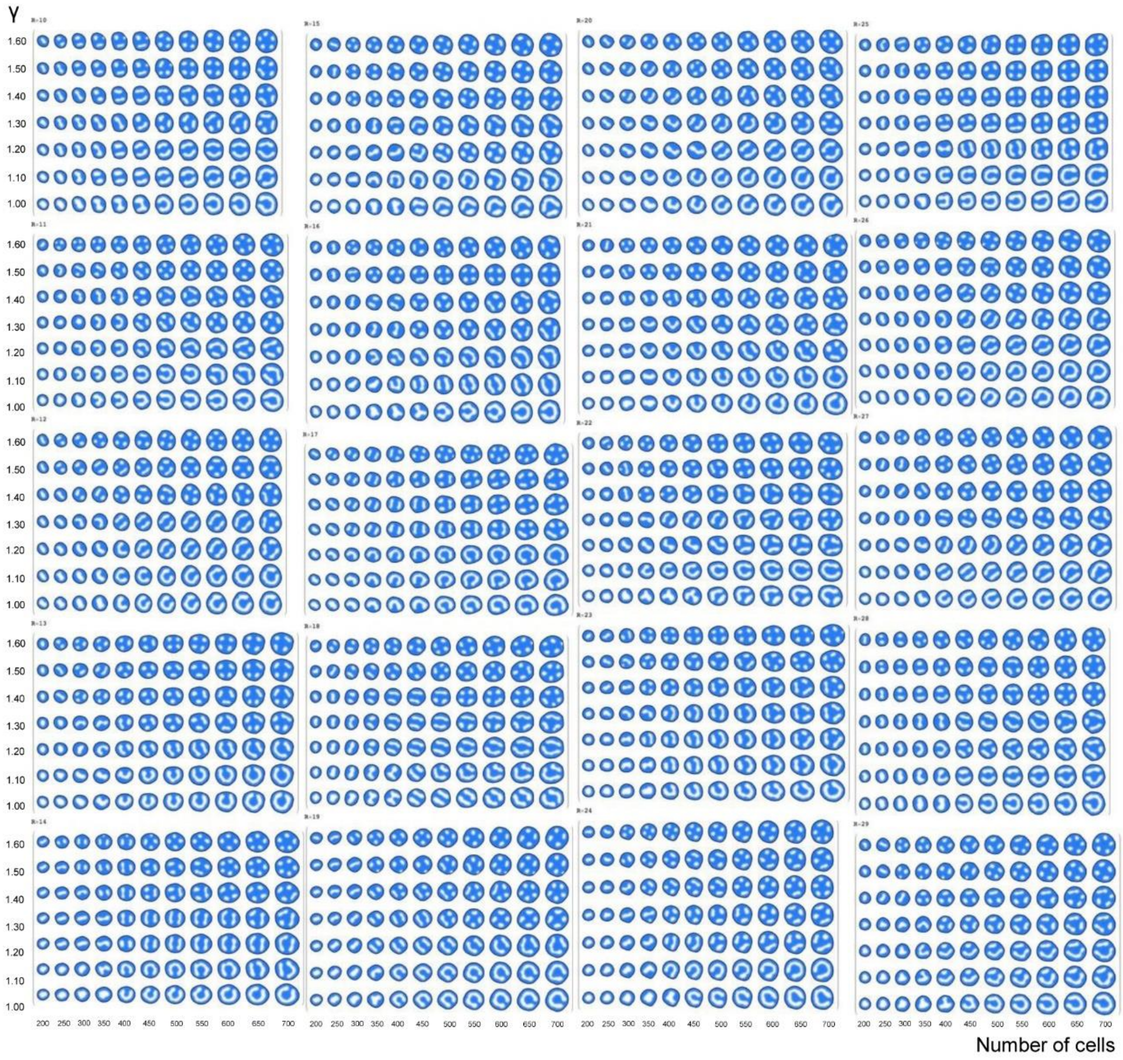

Supplementary Fig. S4. Computational analysis for various γ values in the condition of u_{max} = 1.1u_0. Parameter values are the same as those in Fig. 2E. The results of 20 independent simulations are shown.

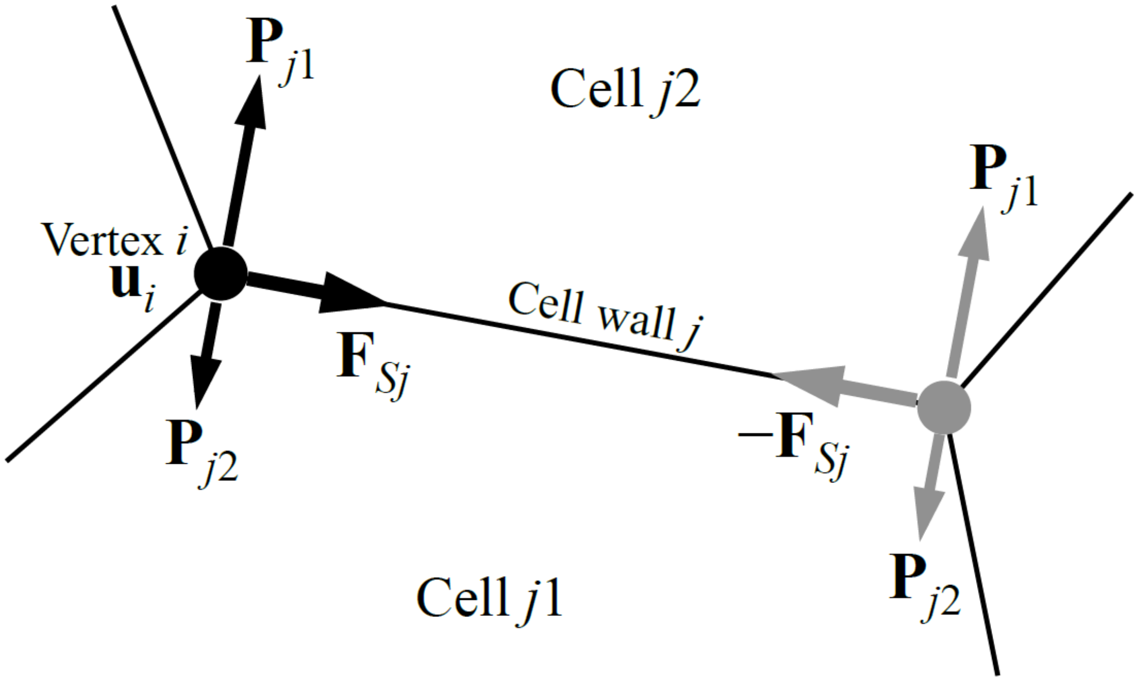

Supplementary Fig. S5. Forces acting on a vertex of cell wall in the simulation.
